# How to Avoid Becoming a Risk Factor of Fecal Incontinence Due to OASIS—A Narrative Review

**DOI:** 10.3390/jcm13175071

**Published:** 2024-08-27

**Authors:** Nikodem Horst

**Affiliations:** 1 Department of General, Colorectal and Oncologic Surgery, Poznan University of Medical Sciences, 61-701 Poznań, Poland; nvh@ump.edu.pl; 2 Obstetrics and Gynaecology Hospital, Poznan University of Medical Sciences, 61-701 Poznań, Poland

**Keywords:** oasis, training, risk factors, sphincter repair, sultan scale

## Abstract

Third- and fourth-degree anal sphincter injuries are among the most severe traumas women can experience during childbirth, often leading to lifelong continence issues. Despite extensive research, current repair techniques are often inadequate, failing to provide long-term efficiency. The repair of OASIS tends to worsen with time as fecal or anal incontinence increases. This article presents the risk factors for primary repair failure differently from those previously described in the literature, specifically focusing on avoidable risk factors related to obstetricians and surgeons who perform OASIS repair. After reviewing the literature, the following risk areas were identified and described: recurrent OASIS, pitfalls of the current WHO classification, surgical techniques, place in which the repair should be performed, surgical training, factors related to low volumes of patients with grade III-IV injuries, timing of the repair, and failure of primary repair.

## 1. Introduction

Third- and fourth-degree anal sphincter injuries are the most severe peripartum traumas in women. The consequences of the trauma, such as continence disorders, often persist throughout the woman’s life [1,2,3,4,5,6,7]. The literature is replete with publications addressing peripartum perineal tears, primary repairs of sphincter injuries, and the various aspects of post-repair anal or fecal incontinence, emphasizing the importance of this issue.

Despite detailed descriptions in the literature, repairs of damaged perineum are characterized by limited effectiveness, including long-term efficacy. The results of repairs of perineal tears, especially those with complete sphincter damage designated as grade III–IV according to the Sultan scale, usually worsen with time [3,4,8,9].

The consequences often include continence disorders and a significant deterioration in quality of life. Rehabilitation, surgery, and medical supervision related to OASIS are frequently required. Despite preventive measures, this few percent of women giving birth are always at risk of and will experience perineal tearing, including severe lacerations. In general, obstetric perineal ruptures are rare, affecting 5.7% of primiparous and 1.5% of multiparous women. [10]. However, grade III–IV tears can be even less common and amount to less than 1% [11].

Although knowledge about risk factors has expanded, the repair techniques remain imperfect, and the results do not meet the current needs of women or doctors. This paper aims to characterize the issue of OASIS diagnosed at the time of the delivery and involving anal sphincters (Sultan grade III–IV), underscoring scenarios where limited practical knowledge or lack of resources can contribute to the risk of fecal and anal incontinence. Such an approach, highlighting the potential role of a physician in the process of primary OASIS treatment and its outcome, is not common in the literature. However, it should be mandatory to deliver appropriate care to patients with childbirth-related sphincter injuries.

## 2. Materials, Methods, and Research Questions

First, all works related to OASIS were found in the PubMed database by searching for the terms “sphincter injury”, “anal sphincter injuries”, “OASI”, “OASIS”, “fecal incontinence”, and “anal incontinence”. Approximately 13,000 works were found. To these results, further filters were applied to retain papers related to the topics of repair and risk related to OASIS within the last ten years. This reduced the number of papers found to about 250. These papers were reviewed, omitting commonly known risk factors of fecal or anal incontinence and focusing solely on factors of OASIS or primary repairs potentially attributable to the doctor. In this way, the following risk areas were identified:

- Recurrent OASIS;
- Pitfalls of the current WHO classification;
- Surgical technique and muscles to be repaired;
- Where to repair perineal lacerations of grade III-IV;
- Surgical training;
- Low patient volumes;
- Delayed primary repair;
- Failure of primary repair.

## 3. Recurrent OASIS

The risk of recurrent OASIS in subsequent natural childbirth is slightly higher when compared with first vaginal delivery [12,13]. The studies highlight that each subsequent perineal injury in a patient correlates with worsened continence [14]. This means that, particularly for patients with third- and fourth-degree perineal injuries, an act as physiological and natural as childbirth can irreversibly decrease their quality of life through fecal incontinence and sexual dysfunction and even influence family planning [13]. It is essential to note that for patients with third- and fourth-degree perineal injuries, cesarean delivery remains the only scientifically recognized and reliable option that does not worsen continence [14,15].

Patients who have already experienced OASIS must have had contributing factors that caused it. Therefore, it is essential to inform patients with previous third- and fourth-degree perineal injuries about the high risk of worsening continence due to natural childbirth after sphincter damage [16], regardless of whether perineal tearing occurs during the subsequent delivery or whether the risk of OASIS is the same [14,17,18]. In any case, the patient should have a decisive say in how the delivery is conducted. It is, however, crucial to realize that reducing the upward trends in cesarean sections should not be at the expense of patients for whom not performing a cesarean section would statistically significantly lead to worsening continence.

Patients who, despite previous third- and fourth-degree sphincter damage, want to give birth naturally must include this fact in their medical documentation for legal reasons, necessarily considering the possibility of worsening continence [14]. Due to the prognostically elevated likelihood of perineal rupture, childbirth for patients who have previously experienced perineal damage and wish to give birth vaginally should take place in facilities with resources available for primary perineal repairs. This type of repair of OASIS is well known to have the best long-term results [19,20].

Given the high expectations for the quality of care in delivering births, combined with often incorrect patient perceptions that childbirth is always physiological, we are increasingly exposed to legal claims due to consequences of perineal tearing—fecal and anal incontinence. With current knowledge, it is necessary to change obstetricians’ approach to patients with risk factors, focusing on individual cases rather than statistics. Once a perineal injury occurs, the patient will be primarily concerned with her condition, not the statistical likelihood of such events. This knowledge requires a dedicated obstetric approach:

1. Patients with third- and fourth-degree perineal injuries should be informed about the risk of reinjury and its consequences, particularly for fecal or anal incontinence. They should be given the freedom to choose their childbirth method, including cesarean section [13,14].
2. Those patients who, after third- and fourth-degree perineal injuries, decide on natural childbirth should—after documenting—give birth in hospitals where doctors are readily available who actually and practically deal with perineal injuries around the clock and ensure continuity of proctological supervision and comprehensive counseling and treatment after these injuries.

This means that in a group of patients with a history of third- and fourth-degree perineal injuries according to the Sultan scale, we can become a risk factor of recurrent sphincter injury and its consequences in the following situations:

- When during the subsequent delivery, incorrect information is provided to the patient about the possible consequences of another natural childbirth;
- When a patient with a history of perineal laceration decides on natural childbirth and there is a lack of a specialist who deals with the surgical treatment of perineal injuries, including care for the patient after this repair; this note concerns patients who have already undergone anal sphincter damage where repairs are technically very demanding.

## 4. Pitfalls of the Current WHO Classification

The commonly used Sultan scale adopted by the WHO and present in scientific research pertains only to anal sphincter injuries [21,22]. It does not include another injury of the levator ani muscle (LAM), especially in the case of operative vaginal deliveries [23]. Since the outcome of perineal tearing for a patient is some form of fecal incontinence, with the current state of knowledge [24], it would be appropriate to review the classification of perineal injuries to include other aspects important for continence, for example, LAM damage, which, according to the literature, may occur in up to 35% of women with OASI [25]. Specific signals for the necessity of changes in classifications may come from studies reporting on injuries of the LAM or the puborectalis muscle, which plays a significant role in fecal continence [25,26].

Currently, there are no widely accepted classifications that allow precise determination of complex perineal injuries (e.g., with LAM damage and avulsion of the vaginal vestibule). Such a classification would enable accurate representation of injuries which often do not fit within the WHO’s standard perineal damage grades I–IV as adapted from the Sultan scale. A more detailed statistical analysis would better identify risk factors for worsening continence.

The existing classification system has limitations, as it tends to oversimplify, with regard to current knowledge, morphologically diverse injuries into just two or four categories (III, IV or IIIa, IIIb, IIIc, IV). This oversimplification does not accurately reflect the complexity of severe injuries occurring during childbirth and does not account for the scope of surgical interventions required for proper management.

Meanwhile, in the current state of knowledge, Sultan’s classification, although it refers to sphincter injuries, certainly does not reflect the scope and complexity of the most severe injuries occurring during childbirth nor does it translate to the range of surgical actions necessary for the proper management of these injuries. In addition, only its two degrees, namely third and fourth, are related to fecal or anal incontinence, although it is commonly used in relation to the very cause of incontinence. This leads to a superficial approach to the topic of perineal injuries—not only for appropriate statistical reasoning and concluding but also for managing perineal injuries. More than simply repairing the damaged sphincter is often required [27]. In the case of complex perineal injuries, a comprehensive and effective repair may require, for example, repair of LAM or levatorplasty and anatomical reconstruction of the perineum to maintain the Parks angle [28].

Everyone involved in primary repairs of severe perineal injuries knows the significance of the imperfections of the commonly used WHO classification: these are diverse injuries, and treating the sphincter damage alone constitutes only part of a comprehensive repair. These observations show that in high degrees (III–IV) of perineal damage, merely performing a repair according to the scope of the injury as commonly classified may be insufficient and lead to worsening continence. The lack of a recommended scale for assessing perineal injuries considering the state of knowledge means that there is no comprehensive ability to accurately describe the severity of perineal damage of LAM, e.g., after operative deliveries, when there is a disruption of the continuity of the rectum without damaging the sphincter complex or avulsion injuries of the vaginal vestibule or rectal avulsion.

A division considering the current state of knowledge should also emphasize the differences between <50 and >50% of external anal sphincter (EAS) injuries (Sultan IIIa, IIIb) and full-length/thickness EAS injuries, regardless of internal anal sphincter (IAS) damage. In the current classification, each incomplete EAS injury requires a different repair technique, usually end-to-end; i.e., the overlapping repair technique may be used only in total EAS disruptions. Due to this imprecision between the commonly used damage scale and the technically justified aspects and possibilities of surgical repair at total or partial EAS disruption, the literature has raised the issue of the repairs’ effectiveness by end-to-end and overlapping techniques [29,30,31].

Thus, we can unwittingly become a risk factor of fecal incontinence by assessing damage only according to the Sultan scale and overlooking, among other things, LAM damage, which requires excellent knowledge of anatomy and surgical techniques for its management [24].

## 5. Surgical Technique and Muscles to Be Repaired

In the current classification of perineal injuries, the literature focuses on repairing the external anal sphincter (EAS) and internal anal sphincter (IAS), emphasizing the importance of correct repair techniques. According to recent papers, LAM injuries are common and should also be repaired due to the effect on fecal incontinence [23,24,26].

There are many works in the professional literature on surgical aspects of primary EAS injury repairs, including end-to-end and overlapping techniques [31,32,33]. These are usually published by specialists or centers specializing in repairing severe perineal damage or by teams routinely dealing with this issue.

The 2006 Cochrane review on the end-to-end technique vs. the overlapping technique did not favor either of these [34], despite concerns related to muscle denervation, atrophy, and scarring, resulting in long-term quality of sphincter injury repairs and fecal incontinence. While practitioners using end-to-end sphincter repair criticized the overlapping technique for necessitating muscle preparation and causing possible further damage, the literature data are unequivocal in supporting the practical outcomes [33]. The overlapping technique is justified only in cases of complete disruption of the sphincters. This technique cannot be questioned when obstetric trauma damage has already caused EAS delamination, creating conditions for the safe application of the overlapping technique without damaging otherwise potentially healthy tissues.

Repair of the EAS should aim to restore the full length of the EAS [35]. A satisfactory long-term effect of the primary repair operation requires repairing both the EAS and IAS, as mere EAS repair with concurrent IAS damage is associated with flatal incontinence and soiling. Therefore, identification of IAS damage is required intraoperatively, and adequate IAS repair must be performed [36,37,38,39].

Repairing LAM injuries is necessary for successful perineal injury repair at birth, as these injuries accompany up to 35% of OASIS cases [25]. The lack of LAM repair contributes to the deepening of incontinence and a significant increase in the rate of incontinence. LAM repair during childbirth is facilitated by easy access (pelvic tissue elasticity), but it can involve substantial bleeding.

A physician repairing perineal injury can become a risk factor for fecal incontinence when repairing the EAS without ensuring the full length of the EAS was performed, when failing to diagnose and repair the IAS, or when failing to diagnose and repair the LAM.

## 6. Where to Repair Perineal Lacerations of Grade III–IV?

The repair of grade III–IV perineal injuries requires a high level of precision and must be performed in an operating room. Adequate patient positioning, good lighting conditions, and regional or general anesthesia, depending on the extent of the injuries, are essential [40,41,42]. Grade III–IV perineal injuries can involve LAM [23], so assistance from a specialist in perineal repair is also preferable.

Birthing beds do not allow for proper patient positioning and exposure of the anorectal area, which is crucial for the visibility of the surgical field and accurate identification of perineal structures. Primary perineal repair is an operation that is particularly encouraging, as it is possible to obtain an excellent view of the perineal tissues that have above-average elasticity because of progesterone. However, physiological childbirth leads to congestion in the perineum and significant intraoperative bleeding, including in muscles. To avoid further iatrogenic damage to the already damaged muscles, the hemostasis should be achieved simultaneously with precise suturing of muscle injuries, using minimally electrosurgical methods or nearly non-electrosurgical methods.

It is technically challenging to repair grade III–IV perineal injuries, even for experienced operators. Comprehensive and professional perineal damage repair goes beyond the commonly understood sphincter repair. Often, only the external anal sphincter (EAS) is addressed using any technique. Attempting to manage perineal injuries on birthing beds can be directly associated with a lack of knowledge about the possible extent of injuries during childbirth. This refers particularly to operative vaginal deliveries [43]. With our current knowledge, the extent of potential soft tissue injuries at birth completely rules out their assessment and management in the conditions of a delivery room and birthing bed. It is essential to note that comprehensive management of perineal injuries includes the recognition and management of internal anal sphincter (IAS) and LAM injuries [44,45,46].

Applying learned perineal injury repair techniques under incorrect resource and equipment conditions, such as outside the operating room or without proper lighting and anesthesia, bares higher risk for the patient. Knowing the appropriate surgical techniques and management for perineal injuries but applying them in an incorrect location other than the operating room should be considered a risk factor for repair failure or incomplete repair, leading to suboptimal outcomes.

## 7. Repair of Perineal Lacerations: Training vs. Reality

Childbirth does not necessarily occur in large centers where several thousand deliveries take place annually and where, statistically, there are dozens of patients with severe obstetric perineal injuries. In such hospitals, frequent damage to perineal structures during childbirth necessitates the availability of teams trained in repairing perineal injuries. However, in smaller hospitals with only a few hundred births a year, after excluding simple type IIIa injuries, it turns out that severe perineal injuries occur in only a few patients annually. Moreover, this small number of patients may be treated by different doctors, resulting in each doctor performing only a few surgeries per year [47]. The rarity and randomness of these injuries significantly hinder the acquisition and maintenance of proficiency in their management.

Despite variations in detailed statistics on the occurrence of perineal injuries, numerous studies have confirmed that the severity of a sphincter injury is linked to worse continence outcomes [48]. However, severe perineal injuries are statistically rare, and yet, they have the most significant long-term effects on patients [3,4,49]. While, in theory, any doctor can manage such injuries, patients benefit most when these operations are performed by a doctor who is experienced in OASIS repair.

Defining the learning curve for perineal repair operations is challenging [50,51]. This is due to the variability of cases and the lack of an immediate, specific effect of the repair. Additionally, training usually only covers the repair of the EAS, ignoring more complex structures like the IAS and LAM. 

Many courses on EAS repair use animal models that are not anatomically related to perineal structures, such as beef tongues [52,53]. Completion of such a course does not guarantee accurate management of a perineal injury, including the IAS and LAM.

The challenging factor in surgical practice managing severe perineal injuries is bleeding from the torn muscles, including EAS and LAM, which can significantly hinder the proper identification of repaired structures [54,55]. These conditions cannot be replicated during training. The lack of practice or training only in simulated conditions results in attempting hemostasis using extensive coagulation (which is usually ineffective) or placing wide sutures, which traumatizes muscles even more. This alters the anatomical conditions in the surgical field, making effective anatomical repair difficult.

Operators may become a risk factor for ineffective repair when the repair of perineal damage is not undertaken routinely or only performed after training in simulated conditions. The overall effectiveness of sphincter repairs after primary repair, as assessed by postoperative ultrasound, is approximately 70% [56]. This is additionally important because, despite anatomical repair, the correct functional effect is often not achieved due to the denervation of the sphincter muscle [57,58]. Professional management of perineal injuries also includes the recognition and repair of IAS and LAM injuries—which are usually not covered at all in repair training.

## 8. Low Volume of Patients

Successful initial repairs of perineal injuries by specialized medical professionals lead to the best long-term outcomes. Most of the published research on this topic comes from large medical centers but a remark must be made that many women give birth in smaller facilities.

The research evaluating physicians’ self-assessment of their skills and knowledge of perineal repair techniques is significant when looking into confidence in identifying and repairing OASIS [51,59,60]. Meanwhile, a common topic regarding skills is even the correct identification of intrapartum perineal injuries, a step that is crucial for proper repair [60]. There can be no correct perineal repair if the repairer does not reconstruct and understand the mechanism of the injury they must manage. Operators handling perineal repairs know that injuries to soft structures rarely look like the illustrations or fixed photographs. This is particularly true for the site of EAS damage, considering its continuity with LAM and its relation to the other pelvic muscles.

Only one study attempted to quantify the extent of training required to practically apply acquired skills of EAS repair to clinical practice, being around 20 repairs, of which 5 were supervised [36,50]. This number of EAS repairs is not enough to manage severe perineal injuries that can involve IAS and LAM. It cannot be ruled out that the outcomes of perineal injury repairs presented in the literature seem acceptable due to the classification of complete and partial sphincter injuries (IIIb) together.

Currently, the number of births is decreasing, and the age of birthing women is increasing, which is a prognostic factor for increasing occurrence of intrapartum perineal injuries. Despite this fact, complex obstetric injuries involving the internal anal sphincter (IAS) and the levator ani muscle (LAM) are rare. Consequently, not all obstetricians or surgeons in every hospital where deliveries occur may have specialized experience in managing these conditions. This obvious fact is only touched upon by a small number of studies that present the actual conditions of perineal injury occurrence; during childbirth, especially in smaller centers, there may be a lack of doctors not only with theoretical skills to manage sphincter injuries but also with a practical lack of these skills due to the rarity of this condition [47]. 

The repair of OASIS is considered effective when it results in minimal inconvenience in terms of maintaining solid and liquid stool continence (note: flatal incontinence is a much lesser inconvenience, though quite common with natural childbirth) [61]. Proper repair of perineal injuries—EAS, IAS, and LAM—requires a deep understanding of the physiology of these structures for maintaining solid, liquid, and flatal continence. Such repairs, even performed by specialists in this field, are characterized by limited effectiveness that tends to decline over the long term despite surgical proficiency in this anatomical area [6,7,62].

Understanding the severity of the injury experienced by a woman is expressed, among other things, by ensuring constant and readily available postoperative care for the patient, including enabling quick visits after leaving the hospital in healthcare systems where they are usually difficult to achieve.

These observations lead to the conclusion that, with the current state of knowledge, the management of intrapartum perineal injuries should be the responsibility of a group of specialists focused on this matter. There should not be a general belief that every obstetrician should perform such repairs without persistent training or based on a course in simulated conditions due to the rarity of Sultan grade III-IV injuries, especially after operative vaginal deliveries. This observation is even more justified if the facility cannot continue care after OASIS in an outpatient setting.

To summarize, a doctor can become a risk factor for fecal incontinence when they perform a few surgeries on difficult perineal injury cases (usually grade IIIb-IV) or if there is no possibility of postoperative care appropriate for the degree of repaired laceration. In such a case, referring the patient to a suitable center should be considered.

## 9. Delayed Primary Repairs

In cases of severe perineal injuries, if there is no experienced surgeon available, rather than expecting the hospital where the patient gave birth to perform the repair, it might be beneficial for the patient to delay the repair and only control the bleeding. After this, awaiting the experienced surgeon or immediate transfer to a center where teams routinely handle perineal repairs, or at least consulting the patient at such a center, is recommended. This should not take more than 12 h. Supporting this approach is the fact that perineal repairs are operations that should be flawlessly performed the first time and that delaying the repair for even 12 h does not worsen the long-term outcomes; this time, in some cases, can be prolonged up to 72 h [19,20,63], which seems to be enough time to obtain, at least in most cases, the consultation.

In summary, in a case where grade III-IV perineal laceration occurs and there is no experienced surgeon available, we can become a risk factor when delaying the time of repair for longer than 12 h. In optimal conditions within this time, the repair should be performed by an attending expert or at a specialized center. However, in some cases, this 12 h time limit can be shifted up to 3 days (72 h).

## 10. Failure of Primary Repair

In case of primary repair failure, there is a rationale to at least try to perform early secondary repair even as late as 21 days from delivery [64]. Early secondary repair is tailored for the management of perineal lacerations when primary repairs fail, which occurs in 2.3% of cases [65]. These operations demand the highest surgical proficiency and may lead to more complications [64]. However, they remain a viable option for patients seeking to avoid the consequences of a failed initial repair, such as incontinence. Therefore, these patients would benefit from being referred for management of primary repair failure.

In case of failure of primary repair, we can become a risk factor for future incontinence if we postpone at least offering a patient an option to perform an early secondary repair. Due to challenging technical details and possible complications, these demanding operations should be performed in a suitable center (or by an experienced team) capable of providing complex postoperative supervision, including outpatient care.

## 11. Conclusions

This paper discusses various risk areas for fecal and anal incontinence that can be attributed to physicians providing childbirth care, including OASIS repair. This perspective could shift the understanding of the problem to recognize that, in some situations, we ourselves may become a factor of ineffective repair. To prevent such a situation, we should pay attention to following risk areas:

1. Patients with a history of grade III–IV perineal damage should be informed about the potential for recurrent perineal tearing during vaginal childbirth, and we should offer them the option of completing the pregnancy via cesarean section.
2. We should remember that the Sultan scale (the current WHO scale) does not reflect all muscles involved in continence; this refers to the levator ani muscle (LAM).
3. The EAS should be repaired in a manner that ensures its full length will be restored. In cases of severe perineal injury, the LAM should always be assessed and repaired if injured.
4. OASIS of grade III and higher should be repaired in the operation theatre to provide the best conditions both for the examination of the OASI and for the repair itself.
5. In case we previously have only repaired perineal lacerations III–IV on animal models, we should ask someone experienced to at least supervise the repair.
6. In case of small patient volumes, lack of resources, or postop supervision, referring the patient to a suitable center should be considered.
7. When perineal laceration III–IV occurs and no experienced surgeon is available, the repair can be delayed up to 12 h. Within these 12 h, the repair should be performed by the attending surgeon or at a specialized center after transferring the patient.
8. Patients with failure of primary repair should be offered an option of having the early secondary repair that can be performed even 21 days post-partum. Due to known complications, failures of primary repairs should be managed in specialized centers that can provide full postoperative patient supervision and care.

The literature suggests that perineal injuries III–IV in many patients are still a challenge when it comes to continence impairment. Performing a primary perineal repair, preferably immediately after delivery (or in its fourth stage), or even delayed by 12 h, ensures the best long-term outcome in terms of continence [19]. Therefore, this should be the standard of care. However, due to the diverse settings of childbirth in different hospitals, acquiring skills for the effective management of perineal injuries and providing comprehensive post-treatment care to patients can be challenging. Proper and thorough repair of perineal injuries requires not only theoretical knowledge of anatomy and repair steps but repeated practical application of these skills. It is unrealistic to expect that every doctor delivering a baby during which a perineal injury occurs will effectively repair third-degree and higher injuries, even if they undergo simulated training. The surgery of perineal lacerations of degree III–IV should not be expected to be performed in any hospital where such damage occurs, so as not to put the patient and a doctor at risk of the consequences of OASIS. For an analogy, upper limb injuries can be considered. Many of them can be managed in any hospital, but the severe ones, or those requiring replantation, usually require management by a dedicated team even though, in theory, limb replantation surgically involves only skilled yet basic surgical procedures. The same should apply to perineal injuries: they should be repaired by the teams trained in managing this area of the body. Note that, unlike the upper limb, the perineum is an unpaired structure, and there is no backup for its function. Therefore, in all cases where there is a risk of suboptimal treatment of OASIS, the patient should be referred to a specialized center.

## Data Availability

Data are available in a publicly accessible repository.
